# The phenomenology of premenstrual syndrome in female medical students: a cross sectional study

**DOI:** 10.4314/pamj.v5i1.56194

**Published:** 2010-04-23

**Authors:** Magdy Hassan Balaha, Mostafa Abd El Monem Amr, Mohammed Saleh Al Moghannum, Nouria Saab Al Muhaidab

**Affiliations:** 1 Department Obstetrics and Gynecology, College of Medicine in Al-Ahsa, King Faisal University, Saudi Arabia,; 2 Department of Neuroscience, College of Medicine in Al-Ahsa, King Faisal University, Saudi Arabia,; 3 Department Obstetrics and Gynecology, Maternity and Children Hospital, Al Ahsa, Saudi Arabia, Hofuf 31982, Saudi Arabia; 4 Department of English Languages, College of Medicine in Al-Ahsa, King Faisal University, Saudi Arabia

**Keywords:** premenstrual syndrome, Saudi Arabia

## Abstract

**Background:**

The premenstrual syndrome (PMS) is particularly common in the younger age groups and, therefore represents a significant public health problem in young girls. This study aims to estimate the prevalence, severity, determinants of premenstrual syndrome (PMS) and its impact among the female medical students in Al-Ahsa, Saudi Arabia.

**Methods:**

This study was performed at the College of Medicine, King Faisal University, Saudi Arabia, from June through December 2009. It included 250 medical students. They filled different questionnaires covering American College of Obstetrics and Gynecology (ACOG) criteria to diagnose PMS, demographic & reproductive factors, physical activity and mental condition. Regression analysis was conducted for all the predictors.

**Results:**

PMS was diagnosed in 35.6% of cases, distributed as 45% mild, 32.6% moderate and 22.4% severe. There were significant trends for older age, rural residence, family income and family history of PMS. The dominant limited activity was concentration in class (48.3%). Limitations of activities were significantly more frequent among severe cases. The prevalence of anxiety and depression was statistically more evident in the PMS group. Regression analysis revealed that, PMS was significantly associated with older age groups, rural residence, lower age at menarche, regularity of menses and family history.

**Conclusion:**

PMS is a common problem in young Saudi students in Al Ahsa. Severe PMS was associated with more impairment of daily activities and psychological distress symptoms. Older student age, rural residence, earlier age of menarche, regular cycles and positive family history are possible risk factors for PMS.

## Background

Premenstrual syndrome (PMS) is used to describe physical, cognitive, affective, and behavioral symptoms that occur cyclically during the luteal phase of the menstrual cycle and resolve quickly at or within a few days of the onset of menstruation [1].

Premenstrual symptoms are experienced by up to 90% of women of child bearing age. A smaller subset meet criteria for premenstrual syndrome (PMS) and less than 10% of them are diagnosed as having premenstrual dysphoric disorder (PMDD) (American Psychiatric Association 2000) [2, 2]. 

The American College of Obstetrics and Gynecology (ACOG) published the diagnostic ten criteria for PMS. It was considered if at least one of the 6 affective and one of the 4 somatic symptoms was reported five days prior to the onset of menses in the three prior menstrual cycles and ceased within 4 days of onset of menses [4].

Various biosocial and psychological causes have been proposed as the cause of the syndrome, including abnormal serotonin function, presence of progesterone, altered endorphin modulation of gonadotrophin secretion, exercise habits, smoking, use of alcohol, altered transcapillary fluid balance and a diet rich in beef or caffeine containing beverages [5]. A myriad of studies has emphasized the importance of examining the cultural context in menstrual experiences. One large multi-country study from 14 cultural groups and women from 10 countries identified different patterns of beliefs regarding interpretations and implications of menstruation reflecting socialization according to demographic variables [6].

Accordingly, while menstruation represents the gir’s entrance to her expected social role as a mature woman the previously mentioned cultural perspectives may have an evident role. From the time of menarche, her family may impose stricter rules on her regarding social behavior. While menstruation may involve positive changes in the social role of the Arab girl, it may also lead to a conflict in attitudes regarding menstruation that may be expressed by negativity and the development of menstrual disorders [7].

The PMS is particularly common in the younger age groups and, therefore represents a significant public health problem in young girls. The Saudi community is undergoing a rapid and economic change. It has a young population structure, with 60% of Saudis fewer than 30 years of age, and 47% under 15. However a little is known about the extent and severity of premenstrual syndromes in Saudi young women. Also, a minority of women with menstrual problems had sought health care and menstruation was revealed to be a highly personal and secretive topic in this population [8-10].

Al-Ahsa province comprises a lot of villages (rural people) around its main four cities (urban people). Hence, the various biological, socioeconomic and lifestyle factors are different from any other area. Cultural features in this part of the world might influence expectations and self-perception of the disease. These include close knit families, prohibition of alcohol, restriction of female smoking, increasing economic level and widespread use of diets rich in calories and caffeinated beverages.

The authors hypothesized that the expected prevalence of PMS in adolescent girls in Al-Ahsa is approximately similar to the rate reported in western countries. The aim of this study included the following: (a) to estimate the prevalence and severity of premenstrual syndrome and (b) to study its determinants and impact among the female medical students in Al-Ahsa, Saudi Arabia.

## Methods

### Participants

This cross-sectional study was conducted at the College of Medicine, Al Ahsa, King Faisal University (KFU), Saudi Arabia over a six months period. All the female students were the target population of the study. Approximately 288 letters and consent forms were distributed to the students. Of these, 271 (94.1%) were returned, signed by participants.

The Students with current medical, psychiatric or gynecological problems were excluded from the study including pregnancies, amenorrhea and significant pelvic pain secondary to a proven or presumptive diagnosis of pelvic inflammatory disease or endometriosis (13 students). Further 8 cases data were incomplete. The final sample was composed of 250 (86.8%) students. This sample completed the ACOG questionnaire, demographic, reproductive and DASS forms.

### Instruments

All the questionnaires were self-reported and were completed by the participants with the aide and observation of a trained researcher about all aspects of the questionnaires.

Socio-demographic and reproductive questionnaire: The questionnaire consisted of 15 questions that included a number of demographic, life style and reproductive variables with combined close and open responses.

ACOG PMS diagnostic criteria: A questionnaire was constructed based on ACOG PMS criteria [1,4] including the following six behavioral and four somatic symptoms; depression, angry outbursts, irritability, anxiety, confusion and social withdrawal breast tenderness, abdominal bloating, headache and swelling of extremities.

Depression Anxiety Stress Scales (DASS): DASS is a set of three self-report scales designed to measure the negative emotional states of depression, anxiety and stress. The essential function of the DASS is to assess the severity of the core symptoms of depression, anxiety and stress. Overall scores are calculated by summing the scores for the relevant items [11]. The Arabic version carried out by Taouk was used [12].

### Instruments

The participants were given liberal verbal explanations plus description letters about the topic and the aim of the study with attached consent forms. Body Mass Index (BMI) was calculated using self-reported data on height and weight. BMI was categorized using cut off points recommended by the National Institute of Health. While four BMI categories were described, women categorized as obese (BMI = 30) were of particular interest in our study.

Participants were deemed to meet the ACOG for PMS if they rated their experience of at least one of the six behavioral symptoms and one of the four somatic symptoms. These symptoms must be recorded in the absence of any therapeutic intervention resulting in social or physical dysfunction and if there was no history of psychiatric and non psychiatric conditions. Symptoms should start during the five days before the menses and relieved within four days of the onset of the menses without recurrence until at least cycle day 13 and are evident for two consecutive cycles.

To estimate the severity of PMS, each item was rated on a scale of 0 “not at all” to 3”extreme”. The highest score of each symptom in the premenstrual period was calculated. Then the total score of PMS was calculated as the sum of the symptom’s score divided by the number of symptoms (mean) and converted to percent. Therefore, the score between 0% -33% represented mild form of PMS, 33% - 66% as moderate and more than 66% was accounted as a severe form of PMS.

ACOG PMS criteria were strictly followed but without the prospective recording for two further cycles due to the socio cultural barriers that interfere with daily reporting of such sensitive issues as menstrual-related symptoms. Data were collected by trained female interviewers. They underwent 6-hour training on the ACOG criteria in 3 separate sessions.

In recording of DASS score items, subjects are asked to use 4-point severity/frequency scales to rate the extent to which they have experienced each state over the premenstrual week ranging from 0 (did not apply to me at all) to 3 (applied to me very much, or most of the time).

### Statistical Analysis

Data were analyzed using the Statistical Package For the Social Sciences, SPSS Inc., Chicago, IL (SPSS version 16). The different sociodemographic, biological and reproductive variables were presented, compared and analyzed using independent t test for continuous and ordinal variables and x^2^ and Fisher exact tests for categorical variables. x^2^ test was used to compare the scales different severities of PMS was used. Variables that were significantly affecting prevalence of PMS on this initial analysis by the x^2 ^test were introduced into the regression analysis model. Different sets of regression analysis were done for each group of variables, then all the significant variables were grouped into final regression analysis to evaluate the role of the different independent variables on the dependant variable; PMS. A p value of < 0.05 was considered significant.

## Results

Of the 250 students approached, PMS was diagnosed in 89 (35.6%) of them using the ACOG criteria. The socio-demographic and reproductive characteristics of the study population are given in Table 1. Among PMS group, the mean age of participants was 20 years. Approximately, two-thirds of subjects were from rural areas with unsatisfactory income and their mean age of menarche was 11.88 years and the mean body mass index was 30.8.

There were significant difference in the socio-demographic data of those with and without PMS such as age, residence, father occupation, family income and family history of PMS. The PMS had a significant trend for older age, rural residence, unstable jobs of the fathers and unsatisfactory family income. Furthermore, PMS group were more likely to report earlier age of menarche, more regular cycles, body mass index greater than 30 and positive family history of PMS. Also, there was a trend for more significant depression and anxiety based on DASS (Table 1).

Using the ACOG criteria, PMS was diagnosed as described before and the total assigned score was categorized into mild, moderate and severe. The frequency distribution of the cases allocated to the three subgroups was; 40 (45%) mild, 29 (32.6%) moderate and 20 (22.4%) severe cases. Premenstrual symptoms were presented in Table 2 in regard to their ranking and severity. The frequency of somatic symptoms were abdominal bloating (75.3) breast tenderness (64.0) and headache (44.9%), whereas the distribution of affective symptoms were confusion (38.2%) irritability (37%), angry outbursts (33.7%), anxiety (33.7%), depression (31.5%) and lastly social withdrawal (25.8%).

The impact of PMS on women’s daily activities was detailed in Table 3.  The activities reported to be limited were concentration in class (48.3%), college attendance (46%), going out of the home (43.8%), daily home chores (41.6%) and homework tasks (36%). Limitations of these activities were significantly more frequent among severe cases (Table 3). As depicted in Table 1, although the prevalence of stress across students with and without PMS did not reveal any significant difference, anxiety and depression scores were statistically more evident in the PMS group. The frequency of depression, anxiety and stress in PMS was presented in Table 3. They were significantly more frequently reported among students with severe PMS.

Regression analysis of the variables that were significantly associated with PMS in the initial x^2 ^testing was done for all sets of predictors by category. All significant predictors were grouped in the final regression analyses presented in Table 4. PMS was significantly associated with older age groups, rural residence, lower age at menarche, regularity of menses and family history.

**Table 1:** Pertinent clinical and socio-demographic characteristics of the study population

		**Cases N =89 No. (%)**	**Non-cases N =161 No. (%)**	**Statistics**	**P value**
**Age in years**:	(mean ±SD)	20.07±0.97	19.75±1.2	T = 2.11	0.036
**Age groups**:	18-19	13(14.6)	55(34.2)	Χ =14.04	0.002
	19-20	21(23.6)	40(24.8)		
	20-21	26(29.2)	26(16.1)		
	21 +	29(32.6)	40(24.8)		
**Residence**:					
	Urban	34(38.2)	102 (63.4)	Χ =14.56	0.0001
	Rural	55(61.8)	59 (36.6)		
**Father educational status**:					
	Less than secondary	39(43.8)	54(33.5)	Χ = 4.93	0.08
	Secondary	19(21.3)	55(34.2)		
	Higher than secondary	31(34.8)	52(32.3)		
**Mother educational status**:					
	Less than secondary	38(42.7)	68(42.4)	Χ = 0.72	0.7
	Secondary	20(22.5)	30(18.6)		
	Higher than secondary	31(34.8)	63(39.0)		
**Father occupational status**:					
	Unstable job	29(32.6)	38(23.6)	Χ = 6.85	0.035
	Employed	29(32.6)	80(49.7)		
	Professional	31(34.8)	43(26.7)		
**Mother occupational status**:					
	Housewife	29(32.6)	47(29.2)	0.314	0.85
	Employed	25(28.1)	48(29.8)		
	Professional	35(39.3)	66(41.0)		
**Mother occupational status**:					
	Unsatisfactory < 6000 SR	33(37.1)	38 (23.6)	Χ =5.09	0.024
	Satisfactory ≥ 6000 SR	56(62.9)	123 (76.4)		
**Age at menarche (years)**:					
	(mean ±SD)	11.8 ± 0.87	12.7 ± 0.7	T = 7.18	0.008
	<= 12	47(52.8)	51(31.7)	Χ = 10.69	0.001
	>12	42 (47.2)	110 (68.3)		
**Body mass index**:					
	(mean ±SD)	30.8 ± 3.9	27.8 ± 5.2	T = 5.07	0.014
	< 30	32(36.0)	96(59.6)	Χ = 12.8	0.000
	≥ 30	57 (64.0)	65 (40.4)		
**Regularity of menstruation**:					
	Irregular	39(43.8)	120(74.5)	Χ = 23.26	0.000
	Regular	50 (56.2)	41(25.5)		
**Duration of menstrual bleeding (days)**:					
	<3	34(38.2)	56(34.8)	Χ = 0.026	0.085
	7-Mar	23(25.8)	43(26.7)		
	>7	32(36.0)	62(38.5)		
**Amount of menstrual bleeding**:					
	Less than average	26(29.2)	45(28)	Χ = 2.51	0.0113
	Average	22(24.7)	55(34.2)		
	More than average	41(48.1)	61(37.9)		
**Family history of PMS**:	(Sister/ mother)				
	Absent	30(33.7)	93(57.7)	Χ = 19.68	0.000
	Present	59(66.3)	68(42.3)		
**Physical activity**					
	Limited	33(37.1)	80 (49.7)	Χ =3.68	0.055
	Unlimited	56(62.9)	81 (50.3)		
**Depression Anxiety Stress Score (DASS)**					
	Depression	24 (27%)	24 (14.9%)	Χ = 5.38	0.021
	Anxiety	39 (40.4%)	45 (28%)	Χ = 6.47	0.01
	Stress	28 (31.5%)	51 (31.7%)	Χ = 0.01	0.97

**Table 2:** ACOG diagnostic criteria of premenstrual syndrome (n =89)

**Severity of PMS**						
	**Total positive**	**Mild (N=40)**	**Moderate (N=29)**	**Severe (N=20)**	**Chi square test**	**P value**
	**No (%)**	**No (%)**	**No (%)**	**No (%)**		
Abdominal bloating	67 (75.3)	25 (62.5)	23 (79.3)	19 (95.0)	Χ =7.16	0.007
Breast tenderness	57 (64.0)	18 (45.0)	20 (69.0)	19 (95.0)	Χ =14.45	0
Headache	40 (44.9)	20 (50.0)	14 (48.3)	6 (30.0)	Χ =0.22	0.638
Confusion	34 (38.2)	5 (12.5)	14 (48.3)	15 (75.0)	Χ =20.2	0
Irritability	33 (37.1)	9 (22.5)	10 (34.5)	14 (70.0)	Χ =10.78	0.001
Social withdrawal	33 (37.1)	5 (12.5)	14 (48.3)	14 (70.0)	Χ =25.07	0
Angry outbursts	30 (33.7)	8 (20.0)	11 (37.9)	11 (55.0)	Χ =11.39	0.001
Anxiety	30 (33.7)	16 (40.0)	10 (34.5)	4 (20.0)	Χ =0.86	0.353
Depression	28 (31.5)	5 (12.5)	12 (41.4)	11 (55.0)	Χ =13.18	0
Swelling of extremities	23 (25.8)	9 (22.5)	8 (27.6)	6 (30.0)	Χ =2.44	0.118

**Table 3:** Impairment of student physical and mental activities in PMS group (n=89)

**Severity of PMS**						
	**Total**	**Mild (N=40)**	**Moderate (N=29)**	**Severe (N=20)**	**Chi square test**	**P value**
	**No (%)**	**No (%)**	**No (%)**	**No (%)**			
**Limited daily activities**						
Concentration in class	43(48.3)	12 (30.0)	17 (58.6)	14 (70.0)	Χ = 9.7	0.002
College attendance	41 (46.1)	12 (30.0)	13 (44.8)	16 (80.0)	Χ = 12.52	0.000
Going out of the home	39 (43.8)	11 (27.5)	15 (51.7)	13 (65.0)	Χ = 8.38	0.004*
Daily home chores	37 (41.6)	10 (25.0)	11 (37.9)	16 (80.0)	Χ = 15.03	0.000
Homework tasks	32 (36.0)	6 (15.0)	14 (48.3)	12 (60.0)	Χ = 13.46	0.000*
**Depression**						
Mild 10-13	13 (14.6%)	3 (7.5%)	5 (17.2%)	5 (25.0%)		
Moderate 14-20	6 (6.7%)	4 (10.0%)	2 (6.9%)	0 (0%)		
Severe 21-27	3 (3.4%)	0 (0%)	0 (0%)	3 (15.0%)	Χ = 8.69	0.003
Extreme 28+	2 (2.2%)	0 (0%)	0 (0%)	2 (10.0%)		
**Anxiety**						
Mild 8-9	14 (15.7%)	7 (17.5%)	3 (10.3%)	4 (20.0%)		
Moderate 10-14	15 (16.9%)	8 (20.0%)	5 (17.2%)	2 (10.0%)		
Severe 15-19	5 (5.6%)	1 (2.5% )	3 (10.3%)	1 (5.0%)	Χ = 6.37	0.012
Extreme 20+	5 (6.7%)	0 (0.0%)	0 (0.0%)	5 (25.0%)		
**Stress**						
Mild 15-18	10 (11.2%)	7 (17.5%)	3 (10.3%)	0 (10.0%)		
Moderate 19-25	10 (9.0%)	3 (7.5%)	6 (20.7%)	1 (5.0%)		
Severe 26-33	4 (4.5%)	0 (0.0%)	2 (6.9%)	2 (10.0%)	Χ = 8.16	0.004
Extreme 34+	4 (4.5%)	0 (0.0%)	0 (0%)	4 (20.0%)		

Items in daily activity were assessed during the premenstrual phase compared to the rest of the month; equal, increased or limited using an activity checklist. Concentration in the class (No of times you were absentminded and the tutor directed you), College attendance (Score of attendance), Going out of the home (No of visits to colleagues or relatives), Daily home activities (Usual home arrangement and sharing in work), Homework tasks (completion of tasks on time)

**Table 4:** Final regression analysis of independent variables significantly associated with PMS

	**B**	**Std. Error**	**Beta**	**t**	**P value**	**95% CI for B**
Higher age group	–.058	.023	–.14	–2.469	.014	–.104 – –.012
Rural residence	.143	.055	.149	2.623	.009	.036 – .251
Unsatisfactory income	–.025	.061	–.024	–.416	.678	–.145 – .094
Lower age at menarche	.213	.055	.22	3.837	0.000	.104 – .322
Higher body mass index	.007	.06	.007	.115	.908	–.111 – .125
Regular menses	.174	.059	.175	2.968	.003	.058 – .289
Family history of PM symptoms	.119	.033	.206	3.633	0.000	.054 – .183
Depression on DASS	.032	.036	.053	.883	.378	–.039 – .104
Anxiety on DASS	.039	.026	.084	1.467	.144	–.013 – .091
Stress on DASS	.022	.028	.046	.800	.425	–.032 – .077
Limited physical activity	–.090	.054	–.093	-1.677	.095	–.195 – .016
R2	.287					
F test	8.697 **					
Constant	–.14					

** = highly significant

## Discussion

This study sheds new light on the phenomenology of premenstrual tension syndrome by estimating the prevalence, symptomatology, physical and mental health impact and identifying the risk factors of the syndrome in a sample of Medical students in Al Ahsa, Saudi Arabia using a structured interview.

The prevalence of PMS in the present study (35.6%) was in accordance with the work of Serfaty et al [13] and Dean et al [14] who reported prevalence of 35%, and 19-30% respectively. Other Western investigator reported higher prevalence of 85% [15]. In Egypt, El-Defrawi et al [16], reported prevalence of 69.6 % while Rasheed and Al-Sowielem [17] in Saudi Arabia, reported a prevalence of 96.6%. A cross cultural investigation conducted in 14 different cultural groups in 10 countries found a lower prevalence rate (23-34%) in nonwestern cultures, while a higher prevalence rate (71-73%) was reported in the western countries [18]. The justification for such difference depends on varied definitions; methods of data collection, sampling technique and the type of study population.

The frequency distribution of the PMS cases as measured by ACOG was allocated as; 45% mild, 32.6% moderate and 22.4% severe cases. This order of frequency was nearly similar to what was reported by Tabassum et al [19]. It was interesting to notice that the frequency of severe PMS was high in our research in contrary to what had been reported by Abuhashim et al [20] and Nisar et al [21] (5.8% and 4.4% respectively). The difference could be due to the recent increase in empowerment and positive gender attitude of young women in Saudi Arabia as a result of rapid development and modernization of the society thus increasing the perception and awareness.

In the current study, the most frequently reported symptom was abdominal bloating (75.3%), which was also reported in previous studies [22]. However, Derman et al [23] reported that the most common symptom was negative affect group as stress and nervousness. This difference may be due to different cultural and socio-demographic variables. Grant stated that individuals in low social ladder may not cope with the stress of the increasingly more challenging environment that may negatively impact physical and psychological well-being [24].

Moreover, the most frequent symptoms in mild and moderate cases were somatic (abdominal bloating, breast tenderness and headache). Whereas, the most frequent symptoms in severe cases included both somatic (abdominal bloating, breast tenderness) and psychological symptoms (confusion, irritability, angry outbursts, social withdrawal and depression). These findings were consistent with Antai et al [25] who showed that somatic symptoms predominated the group with mild - moderate symptoms while mood-related symptoms were predominant in severe conditions.

It was found that 37% of students with PMS reported greater impairment of daily activities; concentration in class (48.3%), attending college (46%), going out of the home (43.8%), daily home chores (42%) and homework tasks (36%). Others reported similar findings [14,25]. This study denoted also that severe degree of PMS was associated with more physical impairment. Montero et al [26] and Tenkir et al [27] reported that academic absence and low achievement was significantly more frequent among college students with severe PMS. Moreover, Yang et al [28] reported that severe menstrual distress was associated with greater burden on mental and physical health than any chronic disease and even comparable to the effect of depression.

In this study, although the prevalence of stress across students with and without PMS did not reveal any significant difference, anxiety and depression scores were statistically more evident in the PMS group. They were significantly more frequently reported among students who reported having severe PMS. The role of stress and major life events has received considerable attention in terms of potential associations with somatic health. Nisar et al [21] reported that the associations between stressful events such as loss of a loved one, recent breakup, work or financial difficulties, and illness and PMS may, therefore, parallel the effect of stressful events on those who are vulnerable to episodes of major depression.

In a nonclinical sample of 91 college students, Portella et al [29] observed that there was a sizable positive correlation between seasonal depressive symptoms and premenstrual symptoms. Perkonigg et al [30] studied 1488 women aged 14-24 years and found that history of traumatic events, history of anxiety disorders and elevated ’daily hassles’ scores were powerful predictors of the development of severe PMS. The difference between these studies and our research may be due to the used tool. We used DASS -21 which had the advantage that: it was psychometrically validated and developed in consideration of cross cultural situations and that our results would reflect the state of these disorders among Saudi young women.

Many factors were analyzed in this study as predictors of PMS using the regression analysis for the age groups, residence, age of menarche, regularity of cycles, and family history.

PMS had a significant association with older age groups in our study. While some authors reported that PMS was increasing with age [31], others failed to find such correlation [32]. Clecknedr-Simth et al [33] found that symptoms were more intense in the 16-18 years group compared to the 13-15 years age group. Bakhshani et al [34] found that the 18-20 years old age group had the highest figures.

Rural residence in our sample was associated with increased PMS. Shershah et al [35] studied PMS in Karachi and found that its prevalence was 33% with the highest figures in lower socioeconomic group living in socially deprived areas. On the contrary, it was reported to be more frequent in young, literate, urban women with more intense symptoms. Despite the level of perception may be high in urban cases, yet the presence of other co-factors, social habits and different living conditions may explain the higher prevalence in rural areas [36]. Our findings of positive associations between PMS and a family history of PMS are similar to some studies done for women in USA and Saudi Arabia [17, 17]. Shared biological and/or psychological factors which may influence expectations and self-awareness may explain mother-daughter dyads.

In the current study, there was an association between regular cycles, younger ages of menarche and development of premenstrual syndrome. These finding were consistent with some previous investigators [2, 15, 20]. On the contrary, others didn’t find any association between PMS and age at menarche [16, 16]. The findings in our study could be explained with the fact that earlier age of menarche and regular cycles are associated with early establishment of ovarian functions and ovulation with fluctuation of steroid hormones in such a young age with less physical and psychological maturity which may lead to PMS manifestations.

### The limitations of this study must also be recognized.

First, our study included a highly selective sample comprising of medical students from one academic institute which will limit the generalizability of the findings. Second, because of the cross-sectional design of the study, we are unable to determine longitudinal relations between any of the studied predictors and outcome and whether they were coexisting or preexisting. Third, Despite the students were included in the study based on absence of medical chronic disorders, yet they were not screened for other possible medical diagnoses when they reported PMS symptoms. Finally, we depend on the retrospective analysis using questionnaires as it was inherently difficult or even impossible to use the prospective approaches. Despite the questions asked were standardized and have been used in other Arab studies [38], we believe that the questionnaire filling is likely to pose some biases, either in the recall or differential classification during the filling with either over or under reporting.

## Conclusion

PMS is a common problem in young students in this part of the world. Severe PMS was associated with more premenstrual symptoms, impairment of daily activities and psychological distress symptoms. Older student age, rural residence, earlier age of menarche, regular cycles and positive family history could be considered as predictors for PMS. Further studies on large sample of population with more preferably prospective approach need to be conducted to confirm these results and to plan out strategies for better detection and management of PMS in young women. The introduction of a reproductive health component into college health education program could help in providing information, education and support to the young students.

## Competing interests

The authors declared they have no competing interests. Also there are no sources of funding.

## Authors’ contribution

**MHB:** Study concept, design, Statistical analysis, Gynecology data discussion manuscript writing, and editing. **MAEMA:** Study concept, design of the questionnaires, psychiatric data discussion, manuscript writing and review. **MSAM:** Evaluation of the students for Inclusion and exclusion criteria, manuscript writing and review. **NSAM:** Female researcher who made awareness of the students, Data collection and helped in manuscript review.

## Acknowledgments

The authors would like to thank Ms. Enas Al Hamam and Ms. Nouf Al Haminy the female technicians at the College of Medicine, Al Ahsa for assistance with cases recruitment and data collection.
